# Female sexual function in bladder cancer: A review of the evidence

**DOI:** 10.1002/bco2.186

**Published:** 2022-09-24

**Authors:** Rebecca Martin, Tessa Renouf, Jeannie Rigby, Shaista Hafeez, Ramesh Thurairaja, Pardeep Kumar, Susanne Cruickshank, Mieke Van‐Hemelrijck

**Affiliations:** ^1^ The Royal Marsden NHS Foundation Trust London UK; ^2^ Action Bladder Cancer Tetbury UK; ^3^ Urology Department, Guys & St Thomas' NHS FT London UK; ^4^ TOUR team Kings College London London UK

**Keywords:** bladder cancer, consequences of cancer, cystectomy, female sexual function, intravesical, living with and beyond cancer, non‐muscle‐invasive bladder cancer, radiotherapy, sexual function, sexual recovery

## Abstract

**Background:**

Bladder cancer (BC) treatments are known to be invasive; nevertheless, research into the long‐term effects is limited and in the context of sexual function often male focussed. Female sexual dysfunction (FSD) has been reported in up to 75% of female patients. This systematic scoping review examines the literature on sexual consequences of BC in female patients.

**Objective:**

This study aimed to systematically evaluate the evidence on female sexual function in BC to identify areas of unmet need and research priorities.

**Evidence Acquisition:**

We performed a critical review of PubMed, PsychMed, CINAHL, MEDLINE and the Cochrane Library in March 2020 according to the Preferred Reporting Items for Systematic Reviews and Meta‐Analysis (PRISMA) *extension for Scoping Reviews* statement following Levac et al. methodology. Identified reports were reviewed according to the Critical Appraisal Skills Programme (CASP) criteria. 45 publications were included.

**Evidence Synthesis:**

There was an inconsistent use of patient‐reported outcome measures (PROMs), with commonly used PROMs having a narrow symptom focus. However, common symptoms emerged: loss of desire, orgasmic disorders, vaginal dryness, dyspareunia, difficult intromission, reduced clitoral sensation, psychological concerns related to diagnosis, fear of contamination and body image. Sexual activity was reduced in most groups, despite women expressing a motivation to retain sexual function. The degree of symptom distress associated with FSD is underreported. Evidence emerged regarding a gap for women in clinician counselling and follow‐up.

**Conclusions:**

The patient's perspective of FSD in BC patients is poorly understood and under‐addressed in clinical practice. There have been very few qualitative studies of FSD in BC. Any intervention designed to address the problem must start with greater understanding of both the patients' and clinicians' perspective.

**Lay Summary:**

We examined the evidence on sexual consequences of BC in women. It is apparent that despite common themes of sexual dysfunction emerging, the problem is poorly understood and addressed in clinical practice.

## 1 INTRODUCTION

Worldwide, bladder cancer (BC) is one of the top 10 most common cancers.^1^, ^2^ BC is more common in men than women; however, in women, it is often more aggressive.^3^, ^4^, ^5^ Therefore, the proportion of women presenting requiring radical treatment is higher.^4^, ^6^, ^7^, ^8^

The acute and long‐term consequences of treatment of both muscle‐invasive (MIBC) and non‐muscle‐invasive BC (NMIBC) can be significant. Despite the knowledge we have about the disease control outcomes of treatments, research into the long‐term effects of BC treatment is limited. Where evidence is available, the focus is the male experience and especially in the context of sexual consequences.^9^, ^10^

Cystectomy is the primary treatment for muscle‐invasive or high‐risk non‐muscle‐invasive disease; for women, this involves removal of the bladder along with the uterus, ovaries and anterior vaginal wall, regional lymph nodes and in many cases urethra, and devascularisation of the clitoris is common.^11^, ^12^, ^13^ Modern surgical techniques coupled with a greater awareness of retaining female sexual function aim to minimise the impact on the neurovascular bundles to preserve clitoral function and in some cases will include vaginal and uterine preservation.^11^, ^12^

For those wishing for organ preservation and with growing evidence of improved oncological outcomes and reduced toxicity, radical radiotherapy may be offered as an alternative to cystectomy in muscle‐invasive disease.^11^, ^14^ In these patients, a commitment to lifelong cystoscopic follow‐up is required.

In non‐muscle‐invasive disease, the treatments are less radical, but patients are required to attend frequent, often invasive investigations and procedures to monitor for disease recurrence.

Female sexual dysfunction (FSD) is a multifactorial condition that is both physical and psychosocial in causation.^15^ It is estimated to have prevalence of 41% in pre‐menopausal females worldwide.^16^ Older age and health factors are consistently reported as contributing factors.^15^, ^17^ FSD can manifest as an arousal, orgasmic or pain disorder that can impact on body image, self‐esteem and intimate relationships and therefore has an impact on quality of life for both the patient and her partner.^18^, ^19^

Sexual rehabilitation is increasingly recognised as an important part of cancer recovery and directly linked to improvements in long‐term quality of life.^20^, ^21^ The James Lind Alliance identified a series of research questions that focus on living with and beyond cancer and specifically how best to manage short‐ and long‐term consequences of cancer treatment to improve experience and minimise the impact on quality of life.^22^ Sexual rehabilitation is an important strategy to support a person's well‐being. However, to date, female sexual function and rehabilitation is better described in the more commonly occurring female cancers such as gynaecological and breast.^17^, ^23^, ^24^ It remains an area of unmet need in females with BC.^9^

The aim of this scoping review is to systematically identify, appraise and synthesise evidence to understand the sexual consequences for females with both MIBC and NMIBC to gain an overview of the research in this field and highlight gaps for future research.

## 2 DESIGN

The National Institute for Health and Care Research (NIHR) Service Delivery and Organisation Research & Development Programme (SDO) recommends scoping reviews to (1) clarify working definitions and conceptual boundaries of a topic, (2) outline what is already known and to identify gaps in knowledge and (3) to perform conceptual analysis to ‘map’ empirical evidence to describe and interpret areas for further research.^25^

This scoping review followed the Arksey and O'Malley (2005) framework for scoping reviews incorporating the Levac et al. (2010) recommendations for additional rigour.^26^, ^27^

Findings are presented using the Preferred Reporting Items for Systematic Reviews and Meta‐Analyses (PRISMA) guidelines extension for scoping reviews.^28^

The primary concept and target population of this review is women who have been treated for BC, specifically focussing on sexual consequences. The review question and aims used an iterative process in consultation with the authors, academic and clinical colleagues. The review seeks to answer the following questions:

- How are sexual function outcomes reported in a female BC population?
- What are the prevalence and common symptoms of sexual dysfunction in female patients with BC?
- How do clinicians address sexual function with patients, and do they use assessment tools?

### 2.1 Search methods

A search strategy was developed using key terms and phrases consistent with BC and sexual health (see Table 1). We searched for studies in March 2020 and repeated in June 2022 in the following databases: Cochrane Library, EBSCO Host (CINAHL, MEDLINE and PsychMed) and PubMed from 2001 onwards until June 2022.

**TABLE 1:** Database search terms

Primary terms	And	And	And
Urinary bladder neoplasm	Cystectomy	Sexual function	Female
Or	Or	Or	Or
Urinary bladder abnormalities	Radical Cystectomy	Sexual dysfunction	Females
Urinary bladder surgery	Anterior exenteration	Sexual health	Woman
Transitional cell carcinoma	Partial cystectomy	Sexual well being	Women
Transitional cell carcinomas	TURBT	Sexual physiology	
Bladder cancer	Transurethral resection of bladder tumour	Sexual pathophysiology	
Bladder carcinoma	Intravesical therapy	Sexual problems	
Bladder malignancy	Intravesical drugs	Sexual difficulty	
Bladder neoplasm	Intravesical BCG	Intimacy	
Bladder tumour	Bacillus Calmette‐Guerin	Sexuality	
Bladder tumours	Intravesical Mitomycin		
Transitional cell cancer	Intravesical erythromycin		
Transitional cell cancers	Intravesical gemcitabine		
Transitional cell malignancies	Chemotherapy		
Transitional cell tumour	Radiotherapy		
Transitional cell tumours	Immunotherapy		

#### 2.1.1 Inclusion criteria

All quantitative, qualitative or mixed‐methods studies and systematic reviews that included measures or themes relating to sexual function.

#### 2.1.2 Exclusion criteria

All studies not available in English and those that excluded a female BC population in the reporting.

#### 2.1.3 Screening procedure

The results were examined by two reviewers (R. M. and T. R.), initially by title, then abstract and finally full paper. Studies, reviews, opinion articles and conference abstracts were included in the screening process; however, no opinion articles or conference abstracts were in the final inclusion.

**FIGURE 1:**
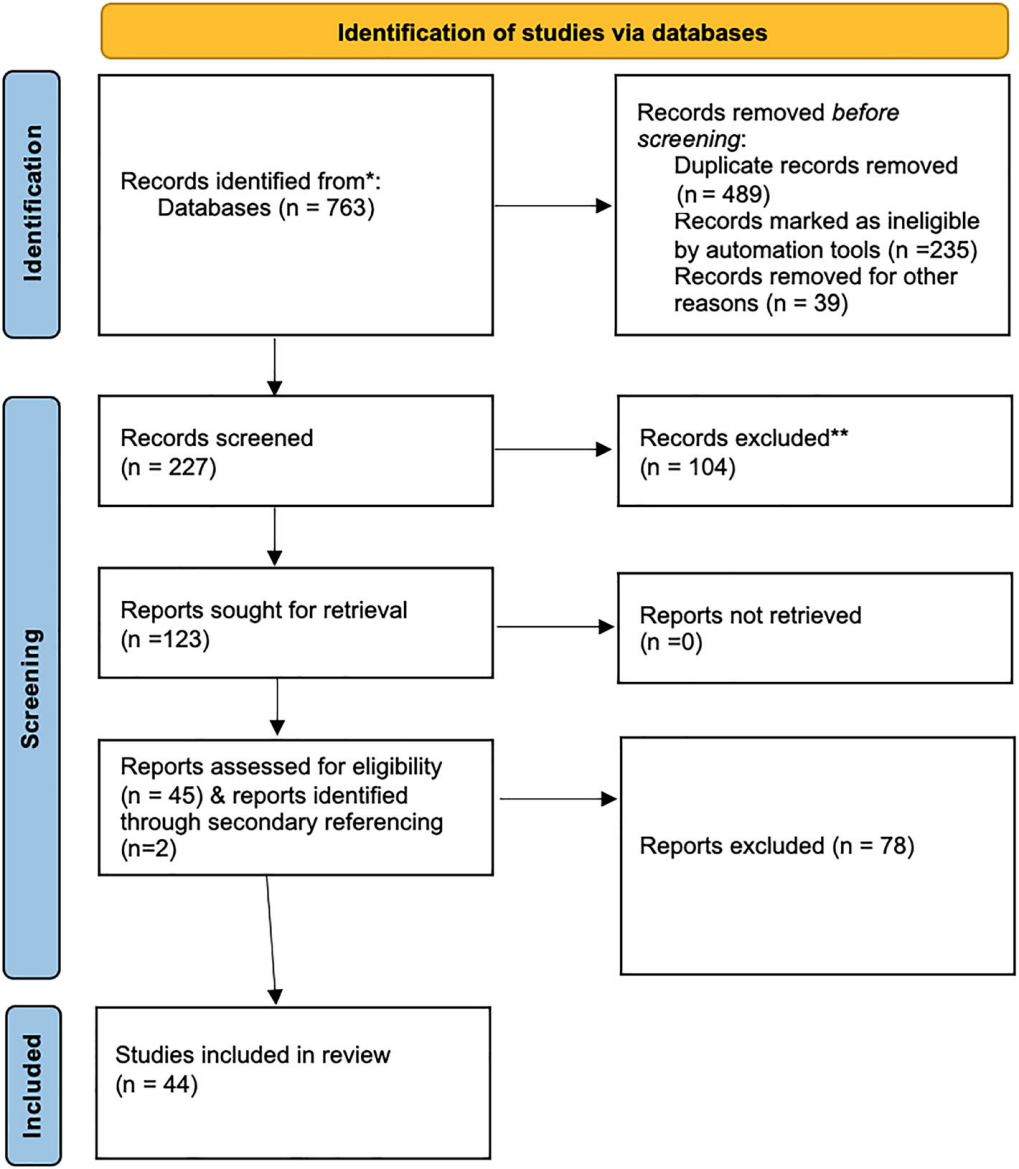
Paper review process

The references of each study were also reviewed to ensure no relevant citations were missed. The study screening process is outlined in Figure 1.

All papers were quality assessed using the relevant Critical Appraisal Skills Programme tool (CASP) and were excluded from the review if they failed to meet two or more CASP criteria.

### 2.2 Data abstraction

The following information was collected from each study at the full‐text stage: type, grade and stage of disease, mean age, information on pre‐treatment and post‐treatment counselling, treatment modality, patient‐reported outcome measures (PROMs) used, type and proportion of sexual dysfunction, sexual interest, sexual enjoyment, intimacy concerns, extent of distress, common themes and reporting of female data sets.

One reviewer performed data extraction, and another checked it. Any discrepancies in data extraction or quality were resolved through discussion.

### 2.3 Synthesis

The search produced a variety of studies, systematic reviews and narrative reviews that are broad in their methodology and findings; therefore, there was no scope for a meta‐analysis.

Due to the breadth of inclusion in this review, the data are presented first by treatment category with PROMs, qualitative studies and systematic/narrative reviews discussed separately. Emerging themes from each category are brought together and analysed in the discussion.

## 3 RESULTS

A total of 763 references were identified; after title screening, 227 went through for abstract review. Of those, 123 references were considered for full‐text analysis. After the full‐text review and removal of duplicates, 45 references met inclusion criteria, including (see Figure 1) 31 primary research and 14 narrative or systematic reviews.

### 3.1 Characteristics of included studies

Of the 31 primary research studies, 3 were qualitative, 26 were quantitative and 2 were mixed‐methods studies using a combination of validated PROMs and/or questionnaires with focus groups. Eleven studies focussed entirely on sexual function, and of these, six looked at female sexual function and the remaining five looking at both male and female outcomes. Of the five studies examining female sexual function, all patients had received a cystectomy. The remaining 20 studies focussed on quality of life or unmet needs (please see Tables 3, 4, 5, 6, 7 for a summary of findings).

Age was often not presented as a standalone variable; rather, it was reported at diagnosis, at time of surgery or at the time of retrospective data collection. The reported ages ranged from 18 to 95 years.

Included in the review are studies looking at different stages and grades of disease; the majority investigate cystectomy, followed by radiotherapy and the non‐muscle‐invasive disease. None of the identified studies looked at patients having systemic immunotherapy.

### 3.2 PROMs

There was a broad range of PROMs used to measure sexual function including the European Organisation for Research and Treatment of Cancer (EORTC) NMIBC C24 and the EORTC BLM30, which were used most frequently for NMIBC and MIBC, respectively. FACT‐BL or the Bladder Cancer Index (BCI) and LENT SOMA were used less frequently. The Female Sexual Function Index was used most frequently as a gender‐specific outcome measure of sexual function. Bespoke surveys were used as commonly as the EORTC modules (*n* = 6). Of the sexual function‐specific studies, all used a sexual function‐specific PROM (see Table 2).

**TABLE 2:** Commonly used PROMs measuring sexual function

PROMs	No. of studies used	Female specific	Questions specific to female sexual function
EORTC BLM30	5		6
EORTC NMIBC C24	5		6
FACT‐BL	2		2
Bladder Cancer Index (BCI)	1		2
LENT SOMA	1	Y	11
FSFI/IFSF	6	Y	19

### 3.3 Radical cystectomy

The majority of available literature focusses on cystectomy, 23 of the 31 studies included (Table 3). Six of the studies compared quality of life and/or functional outcomes with different types of urinary diversion, four studies compared quality of life outcomes in different treatment modalities (these will be reviewed in Section 3.4) and one study compared outcomes between women and men. Six of the cystectomy studies were dedicated to understanding sexual outcomes of surgery in female patients (see Table 3). Eight studies looked at oncological outcomes, quality of life and functional outcomes including sexual function with different types of urinary diversion/reconstruction.^11^, ^12^, ^29^, ^30^, ^31^, ^32^, ^33^, ^34^, ^35^

**TABLE 3:** Cystectomy studies

References	M/F	Age	Sample size	MIBC/NMIBC	Treatment	Study aim	Outcome measure	Key findings: physical concerns	Key findings: psychosexual concerns
Protogerou et al. (2004)	M/F	r50–72	13F IC 5F ONB 12F control	MIBC/HR NMIBC	Cystectomy	QOL	EORTC QLQ‐C30 and bespoke sexual health questionnaire	Loss of desire/libido, orgasmic disorders, vaginal dryness, dyspareunia, difficult intromission and reduced clitoral sensation	Psychological concerns related to diagnosis and body image
Cerruto et al. (2017)	M/F	m64–71	33F IC 15F ONB	MIBC/HR NMIBC	Cystectomy	QOL	EORTC QLQ‐C30 and QLQ‐BLM30	Worsening of sexual function over time with neobladder	
Mohamed et al. (2016)	M/F	m67 r52–82	8F	MIBC/HR NMIBC	Cystectomy	Unmet needs	Bespoke survey	‐ 37.5% <60 and 45.5% >60 affected sexual function Female data: ‐ 25% tx affects body image ‐ 62.5% tx affected sexual function Evidence of age/gender bias	‐ 25% <60 and 18.2% >60 received sexual counselling pre‐op ‐ 37.5% <60 and 13.6% >60 ‘Treatment has affected how they feel about their body’ ‐ All <60s received professional help only 4.5% >60 received help Female data: ‐ 25% had pre‐op sexual counselling ‐ 12.5% received prof support
Mohamed et al. (2014)	M/F	m67 r52–82	8F	MIBC/HR NMIBC	Cystectomy	Unmet needs	Qualitative interviews	Sexual concerns emerged at survivorship stage (6 months post‐op) ‐ 43.3% patients reported sexual function changes (62.5% females). Females reported vaginal dryness and pain	Loss of desire due to body image 25% women were bothered and 12.5% sought prof help
Westerman et al. (2020)	M/F	m68.5	23F	MIBC/HR NMIBC	Cystectomy	SF	EORTC BLM30	High degree of baseline sexual dysfunction in women. Female gender associated with worse SF scores; worse baseline dysfunction	
Gupta et al. (2020)	M/F	NA	NA	MIBC/HR NMIBC	Cystectomy	SF	Questionnaire		‐ 33.6% did not ask female patients about relationships (37% male) ‐ 23.9% did not include partners of female patients in pre‐op counselling (21% male) ‐ 85.5% did not discuss onward referral to sexual or pysch services for female patients (78% male) ‐ 60.8% female patients no routine counselling on baseline sexual dysfunction (20.2% male) ‐ 20% risk of sexual dysfunction (6.5% male) POST‐OP: ‐ 42.6% urologists did not provide routine sexual health counselling to female patients (21.1% male) BARRIERS: ‐ 50.7% older age ‐ 47.1% inadequate time ‐ 37.1% uncertainty of baseline function ‐ 26.4% worried about patient being uncomfortable to talk ‐ 20% lack of knowledge of female dysfunction ‐ 10.7% clinician being uncomfortable ‐ 95% all considered this within their scope of practice
Siracusano et al. (2018)	M/F		33F	MIBC/HR NMIBC	Cystectomy	QOL	EORTC QLQ‐C30 and QLQ‐BLM30	Functional outcomes: women vs. men FU women 52.5 months (men 41.8) ‐ Women had improved outcomes in sexual function than men	
El‐Bahnasawy et al. (2011)	F	m52	38	MIBC/HR NMIBC	Cystectomy	SF	FSFI	Egyptian study: All patients have had female circumcision. Only included sexually active women married to men. Comparison to pre‐op baseline. Questions about relationship status: (? related to SF) ‐ 52% no change in marriage ‐ 39% deteriorated relationship ‐ 8.2% divorced ‐ 26% ceased sexual relations Pre‐op and post‐op satisfaction equivalent ‐ 26% satisfied ‐ 59.2% satisfaction worsened ‐ 14.8% lost all satisfaction Deterioration in FSFI in all domains post‐op Improved SF in orthotopic diversion over IC	
Zippe et al. (2004)	F	m54	27	MIBC/HR NMIBC	Cystectomy	SF	Modified IFSF	80% sexually active pre‐IC and 20% inactive excluded from the study FSFI score reduced from mean 17.4 to 10.6 Significant issues: ‐ 45% diminished/unable to achieve orgasm ‐ 41% dryness ‐ 37% decreased desire ‐ 22% dyspareunia ‐ 48% successful intromission ‐ 48% inability due to reduced clitoral sensation/inability for vaginal penetration No significant difference between the diversions (*no baseline diff in scores between pre‐meno and post‐meno women*)	‐ 52% decreased satisfaction with sex life ‐ 30% partners less desire ‐ Due to concerns about diagnosis and tx
Allareddy et al. (2006)	M/F	m64.4	259 (58F)	MIBC NMIBC	All 82 RC (20 recon and 62 IC) 177 native bladder	QOL	FACT‐G and FACT‐BL	Mean FU time 99.8 months ‐ RC group had worse scores for sexual function	No diffs in long‐term QOL scores between RC and native bladder nor between diversion types
Tyritzis et al. (2013)	M/F	m55.6	70 8F	MIBC/HR NMIBC	Cystectomy	QOL	Bespoke data set	All RC with orthotopic neobladder sparing and sexual organ + vaginal wall sparing. Sexual function evaluated at 12 months ‐ 2 not sexually active ‐ 4 sexually active 66.7% women remained sexually active at 12 months	
Fitch et al. (2010)	M/F	m73.1	22 (9F)	MIBC/HR NMIBC	Cystectomy	QOL	Qualitative interviews	Sex described as uncomfortable Differing levels of importance attached	Issues with body image esp in IC group: ‐ All patients had to ‘make adjustments’ ‐ ‘Revolting’ ‐ ‘Stuck with a bag’ ‐ General improvement in levels of ‘intimacy’ as a consequence of diagnosis
Bhatt et al. (2006)	F	m56.3	13	MIBC/HR NMIBC	Cystectomy	SF	FSFI	6 nerve‐sparing and 7 non‐nerve‐sparing ‐ Overall FSFI minimal decline in nerve‐sparing group (24.5–22.3) ‐ Non‐nerve‐sparing group significant decline (25.0–11.0): ‐ Ultimately 6 of 7 women ceased sexual activity ‐ In the nerve‐sparing cohort, no diffs in lubrication, orgasm and satisfaction	
Henningsohn et al. (2003)	M/F		533 (422 control) C 26% female (75 years) IC 21% (77 years) Mitrofanoff diversion 26% (64 years) neoblad 10% (64 years) RT 27% (81 years)	MIBC/HR NMIBC	Cystectomy	QOL	Bespoke questionnaire	Control group. Cystectomy with recon/IC or RT and control from gen pop Each symptom was scored for severity and a distress level assigned In RC (all diversions) ‐ Reduced orgasm frequency (31–43%) ‐ Reduced orgasmic pleasure (19–32%) ‐ Reduced desire (24–43%) ‐ Sexual limitations (20–36%) ‐ 38% vaginal problems In RT: bowel issues	‐ Symptom distress appeared to reduce with age ‐ All diversions patients symptoms causing greatest (substantial–moderate) distress = reduced frequency of intercourse (34–44%) ‐ 50% of women with feeling of disrupted anatomy were substantially distressed
Booth et al. (2015)	F	m67	41 39 IC 2 pouch 4 ONB	MIBC/HR NMIBC	Cystectomy	SF	FSFI	Pre‐surgery all post‐menopausal ‐ 78% patients sexually active pre‐surgery ‐ 63% not sexually active post‐surgery; of those who remained sexually active (37%) were less frequently sexually active than pre‐op ‐ 41% unchanged sensitivity ‐ 37% decreased ‐ 17% did not answer ‐ 39% unchanged ability to reach orgasm ‐ 44% harder to orgasm ‐ 17% did not answer. Median FSFI score 4.8 (range 1.2–32) Highest scores in satisfaction and lowest in lubrication, pain and orgasm. Statements: ‘I am satisfied with my sex life’, neither agree nor disagree; ‘my sex life is not functioning’, fully agree; and ‘I worry a lot about my sex life’, completely disagree/neither agree nor disagree	‐ 37% reported unchanged desire ‐ 54% decreased desire ‐ 7% did not answer ‐ 61% no concerns with body image affecting sex life ‐ 27% had concerns: stoma bag, lymphoedema and femininity
Gupta et al. (2020)	F	<65 and 65 years	Pre‐op 6 patients and 1 partner Post‐op 16 patients and 2 partners	MIBC/HR NMIBC	Cystectomy	SF	Qualitative	Pre‐ and post‐RC; inclusion of sexually diverse groups	Major themes: ‐ Body image ‐ Psychological impact of BCa/tx ‐ Concerns about impact of RC on sexual function/intimacy and physical changes ‐ Inadequate counselling pre‐/post‐op ‐ Varying degrees of importance on SF ‐ Younger and older participants expressed desire to maintain function
Badawy et al. (2011)	F	m42	78	MIBC/HR NMIBC	Cystectomy	QOL	?	Long‐term FU of females with neobladder—some nerve‐sparing and some not FU mean: 62 months. ? how measured but reported: ‐ 69% patients sexually active pre‐op (65) had sexual dysfunction post‐op incl. ‐ Anorgasmic (23%) ‐ Decreased desire (20%) ‐ Decreased lubrication (15%) ‐ Dyspareunia (11%)	
Gemmill et al. (2010)	M/F	m74	307 97F	MIBC/HR NMIBC	Cystectomy	QOL	COHHRQOL‐O	82.4% BC. Time since surgery mean 9.5 years ‐ 70.4% sexually active prior to surgery ‐ Only 26.7% resumed sexual activity post‐op ‐ 23% satisfied with sexual activity	
Rammant et al. (2022)	M/F	m69	90 (16F)	MIBC/HR NMIBC	Cystectomy	QOL	SCNS‐SF34		Assessment of supportive care needs. Improvement across all domains except for sexual function. Lack of engagement with supportive services
Siracusano et al. (2022)	F	m62.8 ONB and m70.2 IC	37 (9 ONB and 28 IC)	MIBC/HR NMIBC	Cystectomy	QOL	EORTC QLQ‐C30 SF‐36		No robust analysis of SF
Clements et al. (2022)	M/F	m62 ONB and m69 IC	24 ONB and 32 IC	MIBC/HR NMIBC	Cystectomy	QOL	FSFI QLQ‐BLM30 + 12 more	Low scores for sexual function pre‐operatively and limited recovery up to 24 months post. No breakdown of results	
Westerman et al. (2021)	F	m61.7	22	MIBC/HR NMIBC	Cystectomy	SF	Bespoke survey	‐ 77.3% rated vaginal preservation as moderate to very important ‐ Nearly all women noted at least one change in SF, most commonly: ‐ 59.1% dyspareunia ‐ 45% low desire ‐ 42% vaginal dryness ‐ 23% unable to engage in sexual activity ‐ 15% changes in sensation ‐ 9% other (unknown)	‐ 54.5% no pre‐op counselling regarding possible SF changes ‐ 27.3% received some counselling but desired more ‐ 77.8% wish for this be delivered by the care provider

It is reported that up to 70% of female patients with a BC diagnosis experienced a degree of sexual dysfunction.^36^ This was enhanced in patients having cystectomy and urinary diversion of any type.^11^, ^12^, ^30^, ^31^, ^37^, ^38^, ^39^, ^40^ Although findings vary between studies, it is reported that up to 75% of women are sexually inactive following treatment, and in those who report being sexually active following treatment, this declines over time.^11^, ^30^, ^35^, ^41^, ^42^, ^43^, ^44^

Conversely, one study reporting on outcomes in a nerve‐sparing cystectomy cohort found that 66.7% of women remained sexually active at a mean of 12 months post‐operatively.^31^ This finding is consistent with the Bhatt et al.^11^ study that found improved outcomes in sexual function in their nerve‐sparing cohorts. However, Badawy et al. and Clements et al.^32^, ^35^ in their long‐term follow‐up study found significant sexual dysfunction (69%) in a mixed nerve‐sparing and non‐nerve‐sparing cohort.

Common physical symptoms in this cohort include loss of libido, dyspareunia, vaginal dryness, orgasmic disorders and difficult intromission.^11^, ^12^, ^31^, ^32^, ^43^, ^45^, ^46^ Westerman et al.^47^ found that a substantial proportion of patients, male and female, expressed interest in the remaining sexually active following cystectomy; this is consistent with other studies.^48^ Henningsohn et al.^45^ found that the sexual consequences of radical cystectomy were a source of substantial distress to patients. In their 2021 study, Westerman et al.^46^ found that participants placed high importance on vaginal preservation as moderate to very important at 77.3% of women.

### 3.4 Radiotherapy

Four studies were identified that examine radiotherapy or comparing radiotherapy to another treatment modality. Of the included studies, none had a specific female focus, but did extrapolate female data to some extent. Findings are summarised in Table 4. Female participation was between 13% and 27%, and the Fokdal et al.^49^ study is limited to narrative analysis only for the female cohort.

**TABLE 4:** Radiotherapy studies

References	M/F	Age	Sample size	MIBC/NMIBC	Treatment	Study aim	Outcome measure	Key findings
Henningsohn et al. (2003)	M/F	r65–86	Irradiated patients *n* = 48 (13F) Cystectomy *n* = 175 Normal population *n* = 261	MIBC	Cystectomy/RT	QOL	Bespoke questionnaire 125‐ to 139‐item questionnaire	In RT cohort, none of the women in the study were sexually active, all reporting no or low interest in sexual activity during the study period
Fokdal et al. (2004)	M/F	m70	57 RT/66 Con 7F/9F	MIBC	RT	QOL	Telephone structured interview: LENT SOMA	2 women sexually active; 5 lack of desire and 4 lack of satisfaction; 2 women reported that RT had moderate impact on their sex life
Allareddy et al. (2006)	M/F	m64.4	259 (58F)	MIBC NMIBC	All	QOL	FACT‐G and FACT‐BL	82 RC (20 recon and 62 IC) and 177 native bladder. Mean FU time 99.8 months. No diffs in long‐term QOL scores between RC and native bladder nor between diversion types. RC group had worse scores for sexual function
Mak et al. (2016)	M/F	r59–72 at diagnosis	22–25% F	MIBC	All	QOL	EuroQOL EQ‐5D, QLQ‐C30, QLQ‐BLM30, EPIC, CTPS and IOCv2	FU m7 years RC and 9 years RT. F specific items not reported such as dyspareunia and vaginal dryness. RC patients more worried about being intimate and contamination. Body image concerns with RC

Low sexual interest was commonly reported in the studies.^45^, ^49^, ^50^ In comparison, the Fokdal et al.^49^ study found that radiotherapy had a moderate impact on a persons' sex life in approximately 28% of patients and was a more commonly reported problem in females than in males.^49^, ^51^ In the Fokdal et al.^49^ study, a lack of satisfaction in sexual function was reported in 57% of female patients. However, Mak et al.^51^ reported sexual concerns to be less common in patient's having radiotherapy.^51^ Physical symptoms are not well described in these studies.

### 3.5 Non‐muscle‐invasive disease (NMIBC)

With two thirds of BC diagnoses being non‐muscle‐invasive disease, there are disproportionately fewer quality of life studies and even fewer examining sexual function in patients with this disease.^42^, ^52^, ^53^, ^54^, ^55^, ^56^ Six of the studies focussed on NMIBC, none of the studies had a female gender‐specific focus^42^, ^52^, ^53^, ^54^, ^55^ and findings are summarised in Table 5. The female representation in each of these studies was between 12% and 27%, with all but one of the studies providing clear gender extrapolation of the data.^52^ Kowalkowski et al.^54^ carried out a comprehensive mixed‐methods study examining sexual dysfunction in NMIBC; they found that although 68% had interest in sexual relations, only 44% of females in the study were sexually active and up to 63% of females had a degree of sexual dysfunction. Indeed, all of the studies found high scores for sexual dysfunction in the female cohort.^42^, ^52^, ^53^, ^54^, ^55^

**TABLE 5:** Non‐muscle‐invasive studies

References	M/F	Age	Sample size	MIBC/NMIBC	Treatment	Study aim	Outcome measure	Key findings
Madelon et al. (2009)	M/F	m67		NMIBC	Cystoscopy ± intravesical	SF	EORTC BLS24 (NMIBC C24)	54% total M/F reported sexual dysfunction. F17% patient afraid of harming partner
Jung et al. (2020)	M/F	m72	104F	NMIBC	All	QOL	EORTC NMIBC C24	Worse QOL scores for sexual function, sexual enjoyment, sexual probs and sexual intimacy. Worse scores for fear of contamination (lowest in chemo/immuno group)
Siracusano et al. (2018)	M/F	m73	24F	NMIBC	Cystoscopy ± intravesical	QOL	EORTC QLQ‐C30 and QLQ‐BLS24	PROMs measured pre‐tx, mid‐tx and post‐tx; 23% females in study. Low sexual bother scores for both male and female patients (23.3 pre‐probs and 21.0 post‐probs with sexual func; 21.1 pre‐concerns and 16.7 post‐concerns about intimacy). Very low (7.7 pre and 11.1 post) probs with dyspareunia/dryness for females
Schmidt et al. (2015)	M/F		244 (16%F)	NMIBC	Cystoscopy ± intravesical	QOL	Short‐Form 36 (SF‐36) and BCI	FU at diagnosis, 6 months and 12 months; 16% female. Sexual domain deteriorated from diagnosis to 12 months (56.4 to 53.7); 3/4 patients reported sexual bother at diagnosis
Kowalkowski et al. (2014)	M/F	m64.6 survey and m69.1 interview	32F survey 4F interview	NMIBC	Cystoscopy ± intravesical	SF	EORTC BLS24, IIRS, BSI and partner communication score (Porter). Interviews	Demonstrates the relationships between variables such as degree of dysfunction and bother and also relationship and bother ‐ 62.5% vaginal dryness ‐ 23.2% fear of contamination ‐ 55% respondents considered NMIBC had interfered with relationship ‐ 20% shared concerns with partner but ‐ 50% thought it would be helpful to share concerns with partner Interest in sex improved with time since diagnosis. Interviews: ‐ 50% reported sexual dysfunction ‐ 66% reported negative impact on relationship ‐ 33% + fear of contamination
Bolat et al. (2018)	M/F	m57	6F	NMIBC	Cystoscopy ± intravesical	SF	FSFI, EORTC NMIBC C24 and Beck Depression Inventory	FU 1 year post‐diagnosis Mild–moderate sexual dysfunction in women (mean 19.2) ‐ 24% patients not interested in sex ‐ 54% not comfortable with sexual ‘sincerity’ ‐ 20% not sexually satisfied ‐ 57% worried about contaminating partner

Two longitudinal studies were included, for each a different set of PROMs was used (QLQ‐BLS24 and BCI). Both of the studies found that scores for sexual function deteriorated over time.^42^, ^57^ However, Kowalkowski et al.^54^ found that interest in sexual relations increased with increased time since diagnosis. Common physical symptoms included dyspareunia, vaginal dryness and loss of libido.^42^, ^53^, ^54^, ^55^, ^57^

A common theme in all studies was high scores for fear of contamination or fear of causing harm to a partner.^42^, ^52^, ^53^, ^54^, ^55^, ^57^ In the majority of the studies, this concern was reported to be approximately 20–25%; however, in the Bolat et al. (2018) study, this was as high as 57%.^53^

### 3.6 Qualitative studies

From the limited number of qualitative studies (*n* = 3), all concurred high rates of FSD and there is evidence of symptom distress associated with changes in sexual function. The changes in sexual function are reported to be associated with body image and have an impact on intimacy within relationships; the main findings of these studies are summarised in Table 6. Common themes included vaginal dryness, pain, loss of desire due to body image changes associated with a stoma, intimacy changes and low consultation/onward referral rates.^43^, ^48^, ^58^, ^59^ Within the studies, varying degrees of importance were placed on changes to sexual function by the patients, and this was not necessarily age dependent; however, both younger and older participants expressed a motivation to retain sexual function.^43^, ^48^, ^58^

**TABLE 6:** Qualitative studies

References	M/F	Age	Sample size	MIBC/NMIBC	Treatment	Study aim	Outcome measure	Key findings
Mohamed et al. (2014)	M/F	m67 r52–82	30 (8F)	MIBC/HR NMIBC	Cystectomy	Unmet needs	Qualitative interviews	Sexual concerns emerged at survivorship stage (6 months post‐op); 43.3% patients reported sexual function changes (62.5% females). Females reported vaginal dryness, pain and loss of desire due to body image; 25% women were bothered and 12.5% sought prof help
Fitch et al. (2010)	M/F	m73.1	22 (9F)	MIBC/HR NMIBC	Cystectomy	QOL	Qualitative interviews/focus groups	Issues with body image esp in IC group: All patients had to make adjustments. ‘Revolting’. ‘Stuck with a bag’. Sex described as uncomfortable‐differing levels of importance attached and general improvement in levels of ‘intimacy’ as a consequence of diagnosis
Gupta et al. (2020)	F	<65 and >65 years	Pre‐op 6 patients and 1 partner Post‐op 16 patients and 2 partners	MIBC/HR NMIBC	Cystectomy	SF	Qualitative interviews/focus groups	1 homosexual. Major themes: body image and psychological impact of BCa/tx. Concerns about impact of RC on sexual function/intimacy and physical changes. Inadequate counselling pre‐/post‐op. Varying degrees of importance on SF. Younger and older participants expressed desire to maintain function

### 3.7 Systematic reviews

Of the 14 systematic reviews, 9 had a focus on sexual function and 5 of these focussed on female sexual function; of these, 4 had a BC focus. These systematic reviews were looking at female sexual function in radical cystectomy and both with a focus on the sexual consequences of nerve‐sparing surgery; the findings are summarised in Table 7.

**TABLE 7:** Systematic and narrative reviews

Study title	M/F	MIBC/NMIBC	Treatment	Focus
Heyes et al. (2014)	M/F	MIBC/NMIBC	All treatments	QOL
Miranda‐Sousa et al. (2006)	M/F	MIBC/HRNMIBC	Surgery	SF
Smith et al. (2017)	F	MIBC/HRNMIBC	Surgery (not just BC)	QOL
Modh et al. (2014)	M/F	MIBC/HRNMIBC	Surgery	SF
Jensen and Froeding (2015)	F	MIBC	Radiotherapy	SF
Bessa et al. (2020)	M/F	MIBC/NMIBC	All treatments	SF
Avulova and Wittmann (2020)	F	MIBC/HRNMIBC	Surgery	SF
Taarnhoj et al. (2019)	M/F	MIBC	Radiotherapy	QOL
Incrocci and Jensen (2013)	M/F	MIBC	Radiotherapy	SF
Voigt et al. (2019)	F	MIBC/HRNMIBC	Surgery	SF
Paterson et al. (2018)	M/F	MIBC	All treatments	QOL
Arthur et al. (2021)	F	MIBC	All treatments	SF
Donegan and Kingston (2021)	M/F	MIBC	Surgery	QOL
Davis et al. (2022)	F	MIBC	Surgery	SF

Voigt et al.^10^ in their review of the literature looking at simple and radical cystectomy in both benign and cancer conditions suggest that there is a gender bias in urology contributing to the lack of evaluation of outcomes for females. The review is supported with data from a local service evaluation. The authors concluded that despite the high risks of sexual dysfunction with radical cystectomy, few female patients were assessed or counselled accordingly. A secondary conclusion is that consideration of nerve‐sparing techniques should be addressed.

Avulova and Wittmann^38^ conducted a narrative literature review focussing on current knowledge and patient/provider factors contributing to gaps in knowledge. They discussed that as mortality outcomes improve, survivorship and quality of life factors must also improve. Consideration to nerve‐sparing procedures should be offered to female patients. Acknowledgement is given to the complex nature of FSD; however, they concluded that gaps exist in both pre‐operative counselling and post‐operative rehabilitation.^38^

More recently, Davis et al.^60^ reviewed the current evidence on surgical techniques on genitalia and neurovascular preservation and challenge surgeons to consider alternative approaches that preserve female sexual function. The paper makes recommendations for a multi‐disciplinary/multi‐modal approach to management of female sexual function in cystectomy patients, including the surgical, medical and therapy approach to the management of sexual function. A key recommendation is using the 5A's model of patient‐centred care to legitimise and normalise discussion and consultation around the problem.

### 3.8 Clinician views and patient counselling

One study exclusively investigated clinician views on sexual health counselling for patients having cystectomy.^61^ The study found that pre‐operative counselling on sexual function was routine for neither male nor female patients. The counselling of female patients was consistently less likely than for males in most areas including baseline activity and function, risks of sexual dysfunction, nerve‐sparing techniques and post‐operative sexual health. There was a consistent lack of measurement of baseline function more frequently in female patients.^61^ This is congruous with findings from other primary research papers and reviews outlined in this scoping paper.^38^, ^58^

It was determined that the main reasons for the gender disparity included older age of the patient, inadequate time and uncertainty of baseline function.^61^ Gupta et al. recommended that further efforts are aimed at reducing barriers to sexual health counselling and gender disparity.

In their focus group work attached to the 2021 study, Westerman et al.^46^ asked women if and how they wished to be counselled regarding sexual function. They found that greater than half of the women asked had received no pre‐operative counselling with a third more receiving some counselling but wishing for more. The overwhelming majority (77%) wished to receive their counselling on sexual function from their care provider.

Arthur et al.^62^ reviewed the literature on female sexual function in BC recognising the gap in care they developed an educational model for clinicians to use in practice. They recommend the 5A's model as an aide for consultations on the subject and acknowledge that appropriate assessment and referral are essential for high‐quality care.

## 4 DISCUSSION

Female sexual function and therefore dysfunction is complex; it is estimated to affect 41% of the female population worldwide, increasing up to 75% in female patients with BC.^17^, ^36^ Therefore, FSD in BC is a complex problem that requires a complex intervention.

In this review, we have aimed to examine the available evidence on female sexual function in BC, including papers on cystectomy (*n* = 23), radiotherapy (*n* = 4), cystoscopic/intravesical therapy (*n* = 6), along with systematic reviews. Key observations emerged from the literature including inconsistent usage of PROMs, high levels of FSD, fewer studies including radiotherapy or NMIBC cohorts and inconsistent patient counselling on FSD.

The inconsistency in the use of PROMs makes understanding the phenomenon more challenging, particularly the inconsistent use of a female‐specific measure.^9^ Bessa et al. (2020) concluded that more studies were needed to develop, standardise and implement the use of sexual health questionnaires in clinical practice. The existing QOL tools used in the majority of studies have very limited measures on sexual function centred around sexual activity and vaginal dryness and without consideration for the complex issues associated with FSD, disease‐specific concerns nor the psychometric associations of FSD.^9^, ^15^, ^23^ There is ongoing work by the EORTC to validate a sexual health questionnaire standardised for all cancer types for improved assessment in clinical practice as well as research.^63^ The use of standardised units of measure is important for research to ensure validity, reliability and comparability of results.^64^ In some papers in this review, ambiguous terms such as sexual function, vaginal dysfunction, sex life and sexual sincerity were used without describing what they mean.^29^, ^33^, ^53^ Regardless of the lack of PROMs standardisation, there were evident themes across the BC spectrum: loss of desire/libido, orgasmic disorders, vaginal dryness, dyspareunia, difficult intromission, reduced clitoral sensation, psychological concerns related to diagnosis, contamination and body image.

With cystectomy as the primary treatment for muscle‐invasive or BCG refractory high‐risk non‐muscle‐invasive disease and the consequent impact on the female sexual organs, it is unsurprising that the majority of studies in this review focus on this. Current European Association of Urology (EAU) guidelines consider the removal of the sexual organs and anterior vaginal wall to be the standard of care.^13^ A limited number of studies (*n* = 7) have examined sexual function in females having cystectomy often centred around a modified surgical approach.^11^, ^12^, ^29^, ^30^, ^31^, ^32^, ^34^, ^35^, ^65^ Outcomes on sexual function varied but generally reported a better outcome with a modified surgical approach.^11^, ^31^, ^33^ However, it was also found that this diminished over time.^12^, ^29^, ^30^, ^35^ It is clear that across all of the cystectomy patients, there was a decrease in sexual activity and sexual satisfaction post‐operatively.^11^, ^12^, ^29^, ^30^, ^31^, ^32^, ^46^ Common themes from each of the studies include loss of desire/libido, orgasmic disorders, vaginal dryness, dyspareunia, difficult intromission, reduced clitoral sensation, psychological concerns related to diagnosis and body image. There were reports of differing degrees of symptom distress associated with changes in sexual function and intimacy; however, many women expressed a preference to maintain their sexual function.^43^, ^46^, ^48^, ^58^

Of the studies examining radiotherapy, there are a limited number of studies looking at long‐term quality of life following this treatment strategy.^49^, ^51^, ^66^, ^67^ These studies have produced a limited amount of data relating to the impact on female sexual function.^68^ These data suggest some impact on sexual function, more so in females with less sexual satisfaction but less associated distress.^45^, ^49^, ^51^ Further research in this population is needed to better determine this area of unmet need.

In non‐muscle‐invasive disease, several studies have examined the impact of treatment and investigations on sexual function.^54^, ^55^, ^56^ It seems that sexual dysfunction among females was high^54^ and worse than among males.^52^ There was a discrepancy about whether this improved or worsened with time; although with an unpredictable cancer recurrence rate, much of this is likely related to treatment/disease‐related factors.^54^, ^57^ Fear of contamination was a commonly reported concern.^42^, ^52^, ^53^, ^54^, ^55^ Other emerging themes included loss of desire/libido and vaginal dryness.

Most groups report a reduction in female sexual activity following treatment, despite women clearly expressing a motivation to retain sexual function.^45^, ^46^, ^48^, ^49^, ^54^, ^58^ Evidence emerged regarding the gap for women in pre‐treatment sexual counselling and post‐treatment follow‐up.^38^, ^45^, ^46^, ^48^, ^58^ Little is known about clinician comfort and competence to recognise and assess FSD. In a recent study, Gupta et al.^61^ looked at sexual health counselling in cystectomy patients. They found that the counselling of female patients was less consistent than for male patients. Voigt et al. and Davis et al.^10^, ^60^ argue that there is an inherent gender bias in urology that contributes to the lack of evidence. Indeed, as part of this review, several studies only reviewed the male experience and excluded or provided limited detail about the female outcomes.^20^, ^37^, ^51^, ^66^, ^69^, ^70^ Several papers concluded that much more work is needed in this area including pre‐ and post‐operative counselling/rehabilitation, improved measurement and consistency of PROMs to measure sexual function in female patients.^10^, ^20^, ^38^, ^48^, ^58^, ^60^, ^62^

Recent studies by Gupta et al.^48^ and Vencill et al.^71^ conclude that FSD within BC is poorly understood.^48^, ^71^ Arthur et al.^62^ and Westerman et al. make recommendations on ways to manage this in practice; however, this will be challenging to implement without a focus on training and a motivation for culture change. As confirmed by this systematic scoping review, this finding is not isolated to patients with BC, and multiple barriers have been identified to explain this phenomenon across the cancer care setting^23^, ^24^, ^71^: lack of knowledge or confidence on behalf of the treating clinician and patient factors including the lack of confidence or validation to raise concerns.^23^, ^24^ This may lead to a cycle of problems being both underreported and under‐addressed.

### 4.1 Limitations

This review was exploratory and therefore had a wide inclusion criteria. However, the number of studies looking specifically at sexual function in BC was few in number; these were even fewer when limiting to female sexual function. The majority of studies looked at unmet needs or quality of life as a wider issue, and in these studies, sexual function is discussed in the context of overall quality of life, and therefore, findings may be contextual. Several of the studies were conducted greater than 10 years ago. The aims of this review were inclusive of all BC treatment modalities; however, the specific anatomical changes of cystectomy have specific functional sexual consequences. The results of cystectomy studies may need to be distinguished from other treatment types. With a limited number of studies looking at FSD in NMIBC and conflicting findings in the existing studies, we recommend a dedicated systematic review on this subject.

## 5 CONCLUSIONS AND RECOMMENDATIONS

Much of the work on FSD in BC has been undertaken in patients having cystectomy; the findings of this review indicate that FSD across all treatments for BC is common. Inconsistent use of PROMs makes understanding the phenomenon challenging. However, there are common themes across the treatment modalities; there are many presentations of FSD, and the degree of symptom distress is largely under‐explored. Despite a growing momentum into research in this area, there is an evident gap in the measurement and counselling on FSD and the patient's perspective is poorly measured and understood. It is apparent that there is a motivation in female patients to retain sexual function. Better understanding of the female patient perspective of the sexual consequences of BC treatment is needed. There have been very few qualitative studies to help understand the needs of these patients in terms of sexual function. Therefore, any intervention designed to address the problem must start with better understanding of the patients' perspective.

## DISCLOSURE OF INTEREST

None.

## AUTHOR CONTRIBUTIONS

Rebecca Martin was responsible for the conception and design/methodology, data collection, analysis and interpretation, original draft and final approval of the published version. Tessa Renouf was responsible for the design/methodology, data collection, analysis and interpretation, original draft and final approval of the version to be published. Jeannie Rigby was responsible for the critical revision of the article and final approval of the version to be published. Shaista Hafeez was responsible for the conception, critical revision and final approval of the published version. Ramesh Thurairaja was responsible for the critical revision and final approval of the published version. Pardeep Kumar was responsible for the conception, data interpretation, critical revision, final approval of the published version and supervision. Susanne Cruickshank was responsible for the design/methodology, data interpretation, critical revision of the article, final approval of the published version and supervision. Mieke Van‐Hemelrijck was responsible for the conception and design/methodology, data interpretation, critical revision of the article, final approval of the published version and supervision.
